# Predictor of anemia among pregnant women attending antenatal clinics at Hiwot Fana Comprehensive Specialized Hospital, Eastern Ethiopia: a case-control study

**DOI:** 10.1093/inthealth/ihad118

**Published:** 2024-01-16

**Authors:** Tadesse Dufera, Merga Dheresa, Tariku Dingeta, Mezgebu Legesse, Sinetibeb Mesfin, Bikila Balis, Tegenu Balcha

**Affiliations:** School of Public Health, College of Health and Medical Sciences, Haramaya University, Harar, Ethiopia; School of Nursing and Midwifery, College of Health and Medical Sciences, Haramaya University, Harar, Ethiopia; School of Public Health, College of Health and Medical Sciences, Haramaya University, Harar, Ethiopia; School of Medicine, Department of Medical Biochemistry, College of Health and Medical Sciences, Haramaya University, Harar, Ethiopia; School of Nursing and Midwifery, College of Health and Medical Sciences, Haramaya University, Harar, Ethiopia; School of Nursing and Midwifery, College of Health and Medical Sciences, Haramaya University, Harar, Ethiopia; School of Nursing and Midwifery, College of Health and Medical Sciences, Haramaya University, Harar, Ethiopia

**Keywords:** Ethiopia, anemia, determinant, hemoglobin, pregnant women

## Abstract

**Background:**

Anemia during pregnancy is a public health problem and is related to negative birth outcomes, especially in developing countries. The main aim of this study was to assess predictors of anemia among pregnant women attending antenatal clinics at Hiwot Fana Comprehensive Specialized University Hospital, Eastern Ethiopia.

**Methods:**

Unmatched case-control study design was employed among 352 individuals. A face-to-face interview was used to gather data, and each pregnant woman's antenatal care follow-up record cards were reviewed in addition to the interview. EpiData version 3.1 and IBM SPSS version 26 was used for data entry and analysis, respectively. Bivariable and multivariable analyses were conducted to identify predictors of anemia, a p-value of <0.05 was considered a statistically significant association.

**Result:**

The common determinants for anemia in pregnant mothers were: rural residency (AOR = 2.25, 95% CI: 1.14–4.8), no formal education (AOR = 4.4, 95% CI: 1.94–9.9), inter-pregnancy interval (AOR = 2.7, 95% CI: 1.24–5.8), and mid-upper arm circumference (AOR = 5.0, 95% CI: 2.0–12.7).

**Conclusion:**

In this study, the identified determinant factors for anemia were: rural residency, maternal educational status, inter-pregnancy-interval, and mid-upper arm circumference. Therefore, providing health education and promotion for pregnant women regarding anemia by focusing on rural residents and counseling to lengthen their birth spacing is an important task. Moreover, counseling on taking iron supplementation as suggested and consuming a diet rich in iron during antenatal care will be recommended.

## Introduction

Anemia during pregnancy is defined by the WHO as hemoglobin (Hgb) concentrations of less than 11 g/dl for the first and third trimesters and 10.5 g/dl for the second trimester.^1^ Anemia during pregnancy is a public health problem and is related to negative birth outcomes, especially in developing countries.^2^ Anemia can be caused by a variety of factors during pregnancy; it is caused by deficiencies in iron, folate, vitamin B12, and vitamin A, as well as intestinal parasite infections, malaria, and chronic sickness.^3,4^ Fetal anemia, low birth weight, preterm delivery, intrauterine growth restriction, and perinatal mortality were all effects of anemia on both pregnant mothers and their newborns.^5,6^ Anemia during pregnancy is a significant factor in the morbidity and death of pregnant women and newborns in underdeveloped nations.^5,7^

According to the WHO, 32.4 million (38.2%) of women worldwide developed anemia during their pregnancy, with 48% in Southeast Asia and 46.3% in Africa.^8^ Nearly 510 000 maternal deaths are reported each year around the world as a result of childbirth or early postpartum complications. Anemia is responsible for around 20% of maternal deaths, the majority of which occur in underdeveloped nations.^9,10^ The overall prevalence of anemia was 43.3% in sub-Saharan African countries.^11^ In Ethiopia, a pooled prevalence of the systematic review showed that 31.7% of women developed anemia during their pregnancy period; according to this systematic review report the lowest prevalence was observed in the Amahara region (15.9%) and the highest was in the Somali region (56.8%).^12^ Looking at regional variances, the Somali region had the greatest frequency of anemia (60%) followed by the Afar region (45%), and Addis Ababa had the lowest (16.3%).^13^ According to the Ethiopia Demographic and Health Survey 2016, the problem affected 29% of people, and 37.9% of pregnant women in the study area.^13,14^

According to previous findings, undernutrition, low meal frequency, multivitamin deficiency, and a lack of iron folate supplementation during pregnancy are all variables that significantly contribute to maternal anemia.^3,10,15,16^ Mothers with increased gestational age, high parity, and gravidity also have a greater risk of anemia in pregnancy.^7,17,18^ The socioeconomic factors of anemia include rural residency, illiteracy, a large family size, and poor economic status.^19–22^

To address pregnant women's micronutrient insufficiency, the Federal Ministry of Health developed a national nutrition policy. It also set up a system for providing integrated and regular nutritional examinations and interventions such as deworming, folic acid, and iron supplementation.^23,24^ Despite governments’ and stakeholders’ involvement, the anemia problem remains unsolved in Ethiopia, specifically in the study area, and still requires attention. Several studies on the prevalence of anemia in Ethiopia have been carried out, but there is still a gap in identifying the determinant factors and updating the information. In addition to this, there is no documented data on determinants of anemia during pregnancy in the study area. Therefore, this study aimed to assess the determinants of anemia during pregnancy among women attending antenatal clinics at Hiwot Fana Comprehensive Specialized Hospital (HFCSH).

## Methods and Materials

### Study setting and period

HFCSH is located in Harar town, 526 km to the east of Addis Ababa. HFCSH functions as the only referral hospital for the entire eastern part of Ethiopia, Dire Dawa City, the Somali region, and the Harari regional state. It is affiliated with the College of Health and Medical Sciences, Haramaya University, Ethiopia. Currently, the hospital has about 201 beds and 12 case teams to provide referral inpatient and outpatient services to residents of the Harari region and nearby regions. The study was carried out from 23 May 2020 to 23 August 2020.

### Study design and population

An unmatched case-control study was undertaken at the hospital. The study participants were all pregnant mothers receiving antenatal care follow-up at HFCSH. Cases were all pregnant mothers who were attending antenatal care at HFCSH whose Hgb level was <11 g/dl for first and third trimester pregnancy while the Hgb level was <10.5 g/dl for second trimester pregnancy. Controls were all pregnant women who were attending antenatal care at HFCSH whose Hgb level was ≥11 g/dl for first and third trimester pregnancy, as well as ≥10.5 g/dl for second trimester pregnancy. All pregnant women who came for antenatal contact at HFCSH were included in the study. Seriously ill pregnant mothers, who had been on anti-helminthic drugs within the past two weeks, and those who had acute and/or chronic disease-causing anemia were excluded from the study.

### Sample size determination and sampling procedure

To estimate the sample size, a double population proportion formula was employed using Epi Info version 7.2.0.1, with a 95% CI, 80% power, and control-to-case ratio of 2:1. Based on this, the husband's educational status was taken as the main exposure variable with a proportion of 27.3% among cases and 13.5% among controls with OR = 2.4.^25^ Considering a 10% non-response rate, the final sample size was 352 (118 cases and 234 controls). The selection of study participants was made consequently until the required sample size was achieved.

### Data collection instruments and procedures

Information was collected by using a structured questionnaire and reviewing each pregnant woman's antenatal care follow-up records. The questionnaire was modified to fit the local context after being adapted from prior research conducted in Ethiopia and abroad.^25–28^ It consisted of sociodemographic characteristics, knowledge-related, health-related, and maternal dietary status, which were developed in an English-language version and translated into the local language (Afan Oromo and Amharic) before information gathering. It was then translated back into English to maintain consistency.

### Measurements

The WHO definition of anemia in pregnancy was utilized to estimate the hemoglobin cutoff value, pregnant women with hemoglobin levels equal to or above 11 g/dl during their antenatal care services were chosen as controls (non-anemic), while those with Hgb levels less than 11 g/dl were chosen as cases (anemic).^1^ Hemoglobin measurement, malaria attack, and stool examination were taken from maternal antenatal care follow-up record charts. The dietary diversity score was determined using a single 24-h memory, and all liquids and meals taken the day before the research were divided into 10 food groups. Consuming 5 or more out of 10 food groups within 24 h was considered as high food diversity while consuming less than 5 food groups within 24 h was taken as low food diversity.^29^ Besides this, the mid-upper arm circumference (MUAC) was examined using a WHO measuring tape to evaluate malnutrition in pregnant women; a woman was classified as undernourished if her MUAC was less than 23 cm and well nourished if it was greater than or equal to 23 cm.^30^ In this study, cases were assigned ‘1’ and controls were ‘0’.

### Data quality control

Before the actual data gathering, the surveys were pre-tested using 5% of the calculated sample size at Jugal Hospital, and any necessary adjustments were implemented accordingly. During the information-gathering period, two BSc midwives were hired as data collectors and one MSc nurse as supervisor. Training was provided for the data collectors and supervisors on the goal of the study, the clarity of the tools, how to maintain the privacy of the information, and the quality of the data. Intensive supervision was undertaken by the principal investigator, as well as supervisors. Again information taken from medical record cards was cross-checked with participants’ clinical results registered in the laboratory registration book to check their consistency and quality. The supervisors checked the collected data for completeness, accuracy, and consistency.

### Data analysis

Collected information was cleaned, coded, and entered into EpiData version 4.6. SPSS statistical software version 26 was used for analysis. To determine the frequencies, a measure of central tendency, and the variability of the variables used in this study, descriptive statistics were used. Bivariable and multivariable logistic regression analysis was used to identify the association between each independent and dependent variable. Utilizing the variance inflation factor and standard error, multicollinearity was examined to see whether the associated independent variables were correlated. Hosmer-Lemeshow goodness-of-fit and the omnibus test were used to assess the fattiness of the models. To adjust for all potential confounders, a variable with a p-value of 0.25 at 95% CI in the bivariable analysis was added to the multivariable logistic regression analysis. The strength and direction of the association between the independent and dependent variables were then assessed using AOR with a 95% CI and p-values in the multivariable logistic regression analysis. A p-value of 0.05 was then used as the cutoff value to identify the association as statistically significant.

## Result

### Sociodemographic characteristics of study participants

In total, 352 participants (118 cases and 234 controls) took part in the study with a 100% return rate. Participants’ ages ranged from 18 to 40 years with most aged between 24 and 29 (40.1%) years. Around 230 (98.3%) controls and 110 (93.2%) cases were married with 73.3% controls and 40 (33.3%) cases residing in urban areas. Regarding religion, around 116 (49.6%) controls and 92 (78%) cases were Muslim and 39 (16.7%) controls and 57 (48.3%) cases had no formal education (Table 1).

**Table 1.** Sociodemographic characteristics of pregnant women who visited ANCs at Hiwot Fana Specialized University Hospital Eastern Ethiopia, 2020

		Frequency	
Variables	Categories	Cases (118)	Controls (234)
Age	18–23	22(18.6%)	61(26.1%)
	24–29	61(51.7)	80(34.2%)
	30–35	32(27.1)	83(35.5%)
	36–40	3(2.5%)	10(4.3%)
Residence	Urban	40(33.3%)	170(73.3%)
	Rural	80(66.7%)	62(26.7%)
Religion	Orthodox	17(14.4%)	73(31.2%)
	Muslim	92(78%)	116(49.6%)
	Protestant	7(5.9%)	38(16.2%)
	Others	2(1.7%)	7(3.0%)
Mother education	No formal education	57(48.3%)	39(16.7%)
	Have formal education	61(51.7%)	195(83.3%)
Husband education	No formal education	48(40.7%)	52(22.2%)
	Have formal education	70(59.3%)	182(77.8)
Mother occupation	Housewife	97(82.2%)	99(42.3%)
	Government employee	15(12.7%)	107(45.7%)
	Others*	6(5.1%)	28(12%)
Husband occupation	Farmer	89(75.4%)	57(24.4%)
	Government employee	15(12.7%)	125(53.4%)
	Others**	14(11.9%)	52(22.3%)
Income	Unknown	96(81.4%)	87(37.2%)
	2001–3000	2(1.7%)	7(3%)
	>3000	20(16.9%)	140(59.8%)

**Note**: Others: (waaqeffata and catholic), others*: (merchant, students, farmer, and daily worker), others**; (merchant, student, and daily worker).

#### Obstetric-related characteristics

Among the pregnant women who were receiving antenatal care at the hospital, 24.4% of controls and 45% of cases were gravida four and above. The majority of the participants, 65.4% of controls and 51.7% of cases had a birth gap of more than 2 years. Furthermore, 7 (3%) controls and 17 (14.4%) cases had a history of abortion (Table 2).

**Table 2.** Obstetric characteristics of pregnant women who visited ANCs atn Hiwot Fana Specialized University Hospital, Eastern Ethiopia in 2022

		Frequency	
Variables	Categories	Cases	Controls
Gravidity	One	5(4.2%)	28(12.0%)
	2–4	60(50.8%)	149(63.7%)
	>4	53(45.0%)	57(24.3%)
Birth interval	Not delivered	5(4.2%)	28(12.0%)
	≤2 y	52(44.1%)	53(22.6%)
	>2 y	61(51.7%)	153(65.4%)
Gestational age	12–24 wk	22(18.6%)	44(18.8%)
	25–32 wk	47(40.0%)	100(42.7%)
	≥33 wk	49(41.4%)	90(38.5%)
Duration of menstrual flow	≤3	12(10.2%)	25(10.7%)
	4–5	92(78%)	197(84.2%)
	>5	14(11.8%)	12(5.1%)
History of abortion	Yes	8(6.8%)	5(2.1%)
	No	110(93.2%)	229(97.9%)

#### Parasitic infection-related characteristics

The majority of the cases (89%), and almost all of the controls (96.7%), had not had fever in the previous three months. Among antenatal care attendants, 5 (2.1%) controls and 8 (6.8%) cases had had a fever in the last 48 h. Almost three-quarters (72.6%) of cases and 78.8% of controls did not use insecticidal bed net (ITN). Moreover, 5 individuals from controls and 7 from cases had parasitic infections. Among the participants, 21 cases and 10 controls used antimalarial drugs. About 3% of controls and 11% of cases had a history of past medical illness (Table 3).

**Table 3.** Health-related characteristics of pregnant women who visited ANCs at Hiwot Fana Specialized University Hospital, Eastern Ethiopia in 2020

		Frequency	
Variables	Categories	Cases	Controls
Fever last 3 months	Yes	13(11%)	10(4.3%)
	No	105(89%)	224(96.7)
Fever last 48 h	Yes	8(6.8%)	5(2.1%)
	No	110(93.2%)	229(97.9)
Use of ITN	Yes	25(21.2%)	64(27.4%)
	No	93(78.8%)	170(72.6%)
Antimalarial drug use	Yes	21(17.8%)	10(4.3%)
	No	97(82.2%)	224(95.7%)
Deworming	Yes	5(4.2%)	14(6%)
	No	113(95.8%)	220(94%)
History of past medical illness	Yes	13(11%)	7(3%)
	No	105(89%)	227(97%)
Type of medical illness	Malaria	1(7.7%)	1(14.3%)
	Intestinal parasitosis	7(58.8%)	5(71.4%)
	Other	5(38.5%)	1(14.3%)

#### Dietary-related characteristics of the respondent

Nearly two-thirds of controls, 167 (71.4%), and 83 (70.4%) cases consumed foods three or more times a day. About 94 (40.2%) controls and 26 (22%) cases consumed iron-rich foods. The majority of participants, 227 (97.1%) controls and 104 (88.2%) cases, took tea or coffee daily. The majority of pregnant women who received antenatal care, 97 (41.5%) controls and 51 (43.2%) cases, were taking iron supplements. About 28 (23.7%) cases and 46 (19.7%) controls were consuming vitamin A-rich foods (Table 4).

**Table 4.** Dietary-related characteristics of pregnant women who visited ANCs at Hiwot Fana Specialized University Hospital, Eastern Ethiopia in 2020

		Frequency	
Variables	Categories	Cases	Controls
Meal frequency	≤2 per day	35(29.7%)	67(28.6%)
	>2 per day	83(70.3%)	167(71.4)
Iron-rich food	Yes	26(22%)	94(40.2%)
	No	92(78%)	140(59.8%)
Tea/coffee consumption	Yes	104(88.1%)	227(97.0%)
	No	14(11.9%)	7(3.0%)
Fruit and vegetable	Daily	7(5.9%)	32(13.7%)
	Twice	18(15.3%)	32(13.7%)
	Weekly	13(11%)	15(6.4%)
	Very rare	80(67.8%)	155(66.2%)
Used iron supplement	Yes	51(43.2%)	97(41.5%)
	No	67(56.8%)	137(58.5%)
Used iodized salt	Yes	31(26.3%)	82(35.0%)
	No	87(73.7%)	152(65.0%)
Vitamin A	Yes	28(23.7%)	46(19.7%)
	No	90(76.3%)	188(80.3%)
Food diversity	High	15(12.7%)	86(36.8%)
	Low	103(87.3)	148(63.2%)

#### Clinical extract

Regarding the MUAC, out of 352 study participants, 10.3% of controls and 26.3% of cases had a MUAC of less than 23 cm, while 89.7% of controls and 73.7% of cases had a MUAC equal to or greater than 23 cm, respectively. Nearly three-quarters (74.6%) of cases and 214 (91.5%) controls had no intestinal parasite but 3.0% of controls and 14.4% of cases had intestinal parasites. About 5.5% of controls and 11% of cases had signs of bacterial infection.

### Determinants of anemia during pregnancy

To identify independent predictors of anemia, multivariable binary logistic regression analysis was carried out for variables that were candidates at a p-value of less than 0.25 in bivariable analysis. Variables such as husband’s educational status, residency, maternal educational status, use of iron-rich foods, gravidity, birth interval, use of leafy vegetables, iron supplementation, and MUAC were transferred to multivariable binary logistic analysis from the bivariable analysis. Finally, variables such as residency, maternal educational status, inter-pregnancy interval, and MUAC were found to be independent predictors of anemia during pregnancy at a p-value of <0.05 in multivariable analysis.

This study indicated that anemia was nearly three times higher among rural pregnant women than their urban counterparts (AOR = 2.94, 95% CI: 1.22, 7.1). The probability of getting anemia in pregnant mothers who had no formal education was 4.4 times higher compared with those who had received formal education (AOR = 4.4, 95% CI: 1.94–9.9). The odds of developing anemia among pregnant women whose birth interval was less than two years were nearly three times higher than women whose birth interval was greater than two years (AOR = 2.7, 95% CI: 1.24–5.8). According to this study, pregnant women whose MUAC measurement was <23 cm were five times more likely to be anemic compared with their counterparts (AOR = 5.0, 95% CI: 2.14–12.7) (Table 5).

## Discussion

The factors that contribute to the occurrence of anemia must be recognized in order to successfully prevent anemia during pregnancy. Thus, residency, maternal educational status, inter-pregnancy interval, awareness among pregnant women, and MUAC were the variables that were significant predictors of anemia.

In this study, residency was one of the predicting factors of anemia. Women who were living in rural areas were twice as likely to develop anemia compared with those who lived in urban areas. This finding was supported by a study conducted at Adigrat Hospital, northern Ethiopia,^31^ Dera district, northwest Ethiopia,^32^ Bisidimo Hospital, eastern Ethiopia,^10^ Gilgel Gibe dam area, southwest Ethiopia,^33^ and a study conducted in Uganda.^34^ This might be due to pregnant women living in rural areas lacking information about increased nutritional consumption during pregnancy and having limited access to healthcare facilities, making them more exposed to anemia.^35^

**Table 5.** Bivariable and multivariable analysis predictor of anemia among pregnant women visiting ANCs at Hiwot Fana Specialized University Hospital, Eastern Ethiopia in 2020

		Frequency			
Variables	Categories	Cases	Controls	COR (CI 95%)	AOR (CI 95%)
Residency	Urban	40(33.3%)	170(73.3%)	1	1
	Rural	80(66.7%)	62(26.7%)	5.48(1.97–5.72)	**2.25(1.14–4.8)**
Mother's educational status	No formal education	57(48.3%)	39(16.7%)	4.67(2.8–7.7)	**4.43(1.94–9.9)**
	Formal education	61(51.7%)	195(83.3%)	1	1
Husband's educational status	No formal education	48(40.7%)	52(22.2%)	2.40(1.53–3.84)	1.24(0.87–4.56)
	Formal education	70(59.3%)	182(77.8%)	1	1
Birth interval	≤2	52(44.1%)	53(25.7%)	2.46(1.54–4.08)	**2.7(1.24–5.8)**
	>2	61(51.7%)	153(74.3%)	1	1
Gravidity	1	5(4.2%)	28(12%)	1	1
	2–4	60(50.8%)	149(63.7%)	2.25(0.82–6.11)	1.12(0.23–2.43)
	>4	53(45.0%)	57(24.3%)	5.21(1.92–14.53)	2.41(0.98–3.47)
Use iron-rich food	Yes	26(22.0%)	94(40.2%)	1	1
	No	92(78.0%)	140(59.8%)	2.38(1.43–3.92)	1.11(0.67–2.34)
Green leafy vegetable	Yes	54(45.8%)	177(75.6%)	1	1
	No	64(54.2%)	57(24.4%)	3.68(2.34–6.89)	2.14(0.87–2.47)
Iron supplementation	Yes	51(43.2%)	97(41.5%)	1	1
	No	67(56.8%)	137(58.5%)	2.34(1.22–4.64)	0.88(0.68–2.54)
MUAC	<23	31(26.3%)	24(10.3%)	3.12(1.7–5.6)	**5.04(2.14–12.7)**
	≥23	87(73.7%)	210(89.7%)	1	1

AOR: adjusted odds ratio; COR: crude odds ratio; MUAC: mid-upper arm circumference.*p<0.05, **p<0.01, 1 = reference.

One of the key determinants of anemia in pregnant women was the mother's education level. Pregnant women without formal education had four times higher odds of getting anemia than pregnant women with formal education. This finding was supported by studies carried out in different parts of Ethiopia, West Gojjam Zone ,^36^ Benchi Maji Zone,^37^ Woldia Town,^38^ Yrga cheffe health facilities,^39^ and Tanzania.^40^ This might be because women with no formal education did not have sufficient access to information regarding the danger of anemia. Even if they have been advised to take iron tablets and other preventive measures such as consuming iron-rich meals, they may not do so. Furthermore, because education is linked to wealth, illiterate women may not be able to earn enough money to feed themselves during their pregnancy.

This study showed that pregnant women whose pregnancy interval was less than two years were nearly three times more likely to acquire anemia compared with their counterparts. This finding was consistent with studies carried out in North Ethiopia Shire town,^41^ Arba Minch town,^42^ Wollega University Hospital,^43^ Bangladesh,^44^ and India.^20^ The possible explanation for this might be a short inert birth interval that has resulted in a decreased iron store, which may exacerbate the occurrence of anemia in pregnant women.

The likelihood of anemia was five times higher among pregnant women whose MUAC measurement was <23 cm compared with those whose MUAC measurement was >23 cm. The result of this study was strengthened by the study being conducted in the Oromia region,^45^ Dera district, northwest Ethiopia,^32^ Horo Guduru Welega,^46^ West Ethiopia, and Gode town, eastern Ethiopia.^47^ This might be because a MUAC measurement below 23 cm could be an indicator of malnutrition, which is the most common cause of anemia. Moreover, this could be connected to the deleterious impact that protein and other macronutrient deficiencies have on the bioavailability and storage of iron and other hematopoietic nutrients. As a result, the majority of micronutrient deficits are associated with protein-energy malnutrition; to prevent this, micronutrient supplementation is recommended as a routine intervention by the WHO and various local nutritional management guidelines.^48^

### Strengths and limitations of the study

This study utilized a combination of face-to-face interviews and chart review to avoid missing any important variables; the nature of the design helped to establish cause and effect relationships. However, recall and social desirability bias were a common limitation of this study.

### Conclusion

In this study, the identified determinant factors of anemia were: rural residencies, maternal educational status, inter-pregnancy interval, and MUAC. Pregnant women’s awareness of anemia should be increased through strengthened health education and community mobilization on identified determinants of anemia by prioritizing rural women. Enhancing women's education, and increasing family planning accessibility to improve inter-pregnancy interval is mandatory to overcome the problem. Nutritional guidance should be given on consuming foods high in iron and taking iron supplements to prevent anemia in pregnant women. Finally, to reduce sequels of anemia during pregnancy we recommend further community-based studies to explore other risk factors of anemia in pregnancy.

## Supplementary Material

ihad118_Supplemental_File

## Data Availability

All supplemental materials for this article are available from the corresponding authors based on reasonable request.
