# Supplementary material for: Predictor of anemia among pregnant women attending antenatal clinics at Hiwot Fana Comprehensive Specialized Hospital, Eastern Ethiopia: a case-control study
Source: Int Health. 2024 Jan 16;16(4):438–45. doi: 10.1093/inthealth/ihad118 (PMC11218879; doi:10.1093/inthealth/ihad118)
Supplement: ihad118_Supplemental_File [file ihad118_supplemental_file.docx]

Data collection tools

The questionnaire used to collect data for the study done on: Predictor of Anemia among Pregnant Women Attending antenatal Clinics at Hiwot Fana Comprehensive Specialized Hospital, Eastern Ethiopia: a case-control study

**Part I: Socio-demographic and socio-economic Characteristics of the respondents**

| code:_____________ | | | |
| --- | --- | --- | --- |
| S.N | Questions | Responses | **skip** |
| **101** | Age of the mother | ____ in years |  |
| **102** | Residence | 1. Urban 2. Rural |  |
| **103** | Religion | 1. Orthodox 2. Protestant 3. Muslim 4. Other_______ |  |
| **104** | The educational level of the mother | 1. No formal education 2. Have formal education |  |
| **105** | The educational level of the husband | 1. No formal education 2. Have formal education |  |
| **106** | Occupation of the mother | 1. Housewife 2. government employee 3. Merchant 4. Student 5. Farmer 6. Daily worker |  |
| **107** | Occupation of the husband | 1. Employee (GO/NGO) 2. Merchant 3. Student 4. Farmer 5. Daily worker |  |
| **108** | What is your monthly income in Ethiopian birr | 1. unknown 2. 2001-3000 3. Greater than 3000 |  |

**Part II:** 2: **Obstetric-Related Characteristics**

| **S.N** | **Questions** | **Response and Coding** | **Skip** |
| --- | --- | --- | --- |
| **201** | How many times have you got pregnant including the current pregnancy | 1. One time 2. 2-4 3. Above 4 |  |
| **202** | What is the interval between two successive births in the past? | 1. Not given birth yet 2. Less than two years 3. Greater than two years |  |
| **203** | Gestational age of your pregnancy | 1. _____in weeks |  |
| **204** | Duration of menstrual flow | _______ in days |  |
| **205** | Past history of abortion | 1. Yes 2. No |  |

**Part III.** **Parasitic infection and medical status-related questions**

| **S.N** | **Questions** | **Response and Coding** | **Skip** |
| --- | --- | --- | --- |
| **301** | Do you have a fever in the last 3 months | 1. Yes 2. No |  |
| **302** | Do you have a Fever in the last 48Hr | 1. Yes 2. No |  |
| **303** | Have you Used ITN | 1. Yes 2. No |  |
| **304** | Have received Anti-malarial Drug in the past two weeks | 1. Yes 2. No |  |
| **305** | Have you taken Deworming in the past two weeks | 1. Yes 2. No |  |
| **306** | Have you a history of past medical illness | 1. Yes 2. No |  |
| **308** | Type of past medical illness | 1. Malaria 2. Intestinal parasitosis 3. Others ____ |  |

**Part IV. Questions related to the dietary utilization** **of the respondent**

| **S.N** | **Questions** | | **Response and Coding** | **Skip** |
| --- | --- | --- | --- | --- |
| **401** | What is the frequency of meal | | 1. Yes 2. No |  |
| **402** | do you eat Iron-rich food | | 1. Yes 2. No |  |
| **403** | Tea/Coffee consumption | | 1. Yes 2. No |  |
| **404** | How many times do you consume fruit and vegetable per day | | _______ times |  |
| **405** | Do you use an iron supplement | | 1. Yes 2. No |  |
| **406** | Have you used iodized salt | | 1. Yes 2. No |  |
| **407** | Do you use vitamin A | | 1. Yes 2. No |  |
| **408** | **Now, I will ask you about the different food groups you have consumed in the last 24 hours. You will respond by saying ‘Yes’ or ‘No’ for each food group.** | | | |
|  | 1. Foods made from grains (teff, wheat, maize, rice, sorghum, & their products like ‘pasta’, Macaroni, porridge, bread, rice, pasta, or other  2. Pulses (beans, peas, and lentils) Mature beans or peas (fresh or dried seed), lentils or bean/pea products  3. Dark green leafy vegetables including wild/foraged leaves, Vitamin A-rich vegetables (roots and tubers Pumpkin, carrots, sweet potatoes that are  4. Fruits (orange, banana, etc.)  5. White roots and tubers and plantains  6. Meat and poultry(beef, goat, chicken)  7. Eggs  8. Fish and seafood  9. Milk and milk products (cheese, yogurt, or other milk products)  10. Nuts and seeds( Any tree nut, groundnut/peanut or certain seeds, or nut/seed “butter”) | | |  |
|  | **Part V. question used for clinical extraction** | | |  |
| **501** | Stool examination | 1. no finding 2. Ova parasite seen 3. any sign of bacterial infection | |  |
| **502** | MUAC | ______ in numbers | |  |
